# Supplementary material for: Detecting joint attention events in mother-infant dyads: Sharing looks cannot be reliably identified by naïve third-party observers
Source: PLoS One. 2021 Jul 23;16(7):e0255241. doi: 10.1371/journal.pone.0255241 (PMC8301644; doi:10.1371/journal.pone.0255241)
Supplement: S1 Table — (DOCX) [file pone.0255241.s001.docx]

**S1 Table.** Confusion Matrix between how mothers assigned their infants’ looks and how raters assigned the looks for which they had high agreement*:*

|  |  | **Raters** | | |
| --- | --- | --- | --- | --- |
|  |  | **Checking** | **Orienting** | **Sharing** |
| **Mothers** | **Checking** | 1 | 1 | 1 |
|  | **Orienting** | 0 | 0 | 1 |
|  | **Sharing** | 1 | 0 | 2 |
|  | **Unknown** | 0 | 1 | 1 |
